# Distinctive gene expression in the reduced first thoracic legs of a nymphalid butterfly

**DOI:** 10.1111/imb.70045

**Published:** 2026-06-02

**Authors:** Asia E. Hoile, Peter O. Mulhair, Peter W. H. Holland

**Affiliations:** ^1^ Department of Biology University of Oxford Oxford UK; ^2^ Institute of Infection, Veterinary and Ecological Sciences, University of Liverpool Liverpool UK

**Keywords:** chemosensation, homeobox, Meadow Brown, opsin

## Abstract

Nymphalid butterflies have unique leg morphology among Lepidoptera: they are the only family with greatly reduced forelegs (T1) in adults of both sexes, which are not used for walking. Previous studies have suggested that T1 legs may have chemosensory functions. To investigate which genes underpin this biology, we undertook a differential gene expression analysis in female *Maniola jurtina*. We find that nymphalid T1 legs have a distinct transcriptomic profile to T2 and T3 legs and also distinct from sensory palps. We find over 250 genes commonly expressed in nymphalid T1 legs and palps, but not in T2 legs. Despite this overlap, T1 legs are still more similar to T2 and T3 legs in their gene expression profiles than they are to palps. Genes expressed in common between palps and T1 legs include one encoding a trypsin‐domain protein descendent from a nymphalid‐specific duplication and several others involved in sensory functions, including genes with putative chemosensory roles. A blue‐sensitive opsin gene is specifically expressed in T1 legs. Our findings indicate clear transcriptomic differences between T1 legs and walking legs in *M. jurtina*, pointing to the functional basis of these differences, including minor acquisition of palp‐like gene expression.

## Graphical abstract

- Transcriptome analysis reveals that the small non‐walking front legs of a butterfly have distinct gene expression to the four walking legs.
- Reduced front legs express a gene with putative mechanosensory roles, a blue‐sensitive opsin and two homeobox genes.
- Reduced front legs express some genes in common with sensory palps, including chemosensory and phototransduction genes and a member of a gene family duplicated in Nymphalidae.

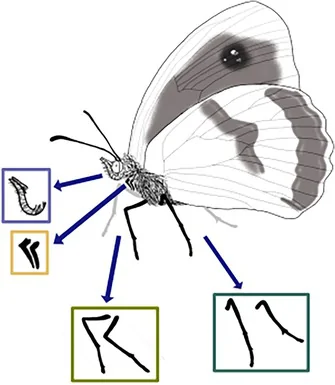

## INTRODUCTION

Although the majority of adult Lepidoptera typically walk on all six of their legs, two taxonomic families are characterised by greatly reduced forelegs (T1 or prothoracic legs), meaning they only walk using the legs of the second and third thoracic segments (T2 and T3 respectively) (Wolfe et al., 2011). These two families, Nymphalidae and Riodinidae, likely evolved reduced forelimbs in parallel, and it is notable that reduced T1 legs are observed in both sexes in Nymphalidae, but only in male riodinids (Wolfe et al., 2011). Despite this, the T1 legs in nymphalid females retain all five tarsal segments, while in males the post‐tarsus is never present (Fox, 1966).

The Riodinidae are a sister lineage to the Lycaenidae, and together these lineages are sister to Nymphalidae (Kawahara et al., 2023; Wolfe et al., 2011). A slight reduction has been observed in other butterfly species, and the degree of reduction of T1 legs and the differences in reduction between sexes have traditionally been among the main characteristics used to define major butterfly taxa (Fox, 1966). Hesperidae and Papilionoidea have minimal reduction, with T1 legs being only slightly shorter than T2 and T3 legs. Lycaenidae have forelegs distinctly smaller than T2 and T3 legs; however, all three families use all three sets of legs for walking (Fox, 1966).

It has been suggested that the T1 legs of nymphalids evolved novel sensory functions, subsequently losing their walking function (Wolfe et al., 2011). For example, tarsal sensilla on T1 are implicated in host plant recognition prior to oviposition (Baur et al., 1998; Calvert & Hanson, 1983), and the physiological reactions of sensilla to plants differ between T1 and T2 or T3. Furthermore, nymphalid T1 legs demonstrate a negative reaction to sugar solution, whereas a positive reaction is observed from T2 and T3 legs (Fox, 1966). This difference is not observed in the Pieridae family, where both T1 and T2 legs demonstrate a positive reaction to a sugar solution (Fox, 1966). The number of putative sugar transport genes expanded throughout the evolution of Lepidoptera, giving potential for specialisation of roles (Hoile et al., 2025). Similarly, previous work found a link between gene duplication in key sensory genes, the gustatory receptors responsible primarily for taste perception and the localised expression of these paralogs in the legs of *Heliconius* butterflies (Briscoe et al., 2013). This leg specific expression was more pronounced in females, which, accompanied by sexual dimorphism in the number of gustatory sensilla present on the T1 legs, is thought to play an important role in oviposition behaviour (Renwick & Chew, 1994).

Legs are not the only appendages with sensory roles in insects. Palps are small, segmented appendages near the mouthparts of insects (Kristensen & Skalski, 1998; Scoble, 1995). In Lepidoptera, the term ‘palps’ typically refer to labial palps, paired structures that form part of the labium and hence part of the last (most posterior) head segment. Lepidopteran labial palps have roles in chemoreception, in tactile sensing to guide feeding behaviour and, in some cases, the protection of the coiled proboscis (Diiak et al., 2023; Lee et al., 1985; Myers, 1969). Maxillary palps, associated with the segment immediately anterior to the labium, are typically reduced or vestigial in Lepidoptera due to the evolution of a proboscis, which is specialised for nectar feeding; an exception is found in the families Micropterigidae, Agathiphagidae and Heterobathmiidae which lack the proboscis (Diiak et al., 2023; Krenn, 2010).

Arthropod legs and mouthpart‐associated palps are considered to be serially homologous structures, meaning they have evolved from a common ancestral appendage type (Hughes & Kaufman, 2002; Panganiban et al., 1997). Consistent with proposed serial homology, genes necessary for appendage development, and typically expressed in arthropod legs, are also expressed in mouthpart‐associated palps. For example, in situ hybridisation revealed that the *Distal*‐*less* (*Dll*) gene is expressed in a ‘sock‐and‐ring’ pattern in the developing walking legs of the butterfly *Junonia coenia*, and in a subtly different ‘sock‐only’ pattern in developing labial palps (Panganiban et al., 1994). Gene expression similarities are observed in other arthropods, including expression of *Distal*‐*less* (*Dll*), *homothorax* (*hth*) and *dachshund* (*dac*) in Crustacea and Chelicerata (Abzhanov & Kaufman, 2000; Barnett & Thomas, 2013; Sharma et al., 2012). Studies in *Drosophila melanogaster* and *Oncopeltus fasciatus* have identified Hox genes as responsible for the specification of these different head structures; in particular the *proboscipedia* (*pb*), *Deformed* (*Dfd*) and *Sex combs reduced* (*Scr*) genes (Angelini et al., 2005; Aplin & Kaufman, 1997; Hughes & Kaufman, 2002).

The above studies have demonstrated shared evolutionary origin and some degree of developmental similarity between legs and palps, but they clearly evolved into very distinct structures in insects: in essence, legs are primarily for walking and palps are for sensing (Briscoe et al., 2013; Lee et al., 1985). In this context, the dramatic size reduction of T1 legs in nymphalid butterflies is intriguing; they are no longer used for walking and there is evidence for a novel sensory role (Baur et al., 1998; Briscoe et al., 2013; Calvert & Hanson, 1983; Myers, 1969). However, morphologically, they do not resemble palps and appear instead as small leg‐like structures on the T1 segment (Fox, 1966; Wolfe et al., 2011). We wished to investigate whether nymphalid T1 legs resemble T2 and T3 legs (walking legs) in gene expression profile, despite their morphological reduction, or whether they express a distinctive set of genes. We also ask whether nymphalid T1 legs have similarity in transcriptomic expression to labial palps in nymphalids, thereby testing if they have co‐opted biochemical or physiological characteristics of palps. Finally, we asked whether any transcriptomic expression differences between T1 and walking legs were related to genes that had arisen through genetic novelty originating during nymphalid evolution; for example, whether new genes may have evolved specifically for roles in the T1 legs. We chose to focus on adults of *Maniola jurtina* (Meadow Brown), an abundant European grass‐feeding butterfly (family Nymphalidae, subfamily Satyrinae).

## EXPERIMENTAL PROCEDURES

### Sample collection and RNA sequencing

*Maniola jurtina* (Meadow Brown) individuals were collected between 17 June 2024 and 20 August 2024 at Wytham Woods, Oxford (UK grid reference SP 468085). Specimens were stored at −70°C and dissected on dry ice while frozen. Palps, T1, T2 and T3 legs were removed and pooled from multiple individuals into three biological replicates, ensuring internal consistency within a batch. Batch MBF1 comprised 6 females collected on 17 June 2024, batch MBFB comprised 3 females collected on 17 June 2024 and 12 collected on 12 August 2024, and batch MBFD comprised 6 females collected 20 August 2024 (Table S1). One batch comprising 15 males was also sequenced but not analysed; data are deposited to facilitate use by other researchers (Table S1). RNA was extracted from pooled samples using the RNAeasy micro kit (Qiagen) and eluted in 20 μL of ultrapure water, and further pooled if RNA yield was low. RNA sequencing was performed on replicates using the Illumina NovaSeq X Plus Series (PE150) Sequencing System (Novogene) at a coverage of 20 M paired‐end 150 bp reads per pooled sample.

### Differential gene expression analyses

Reads were mapped to the *M. jurtina* reference genome ilManJurt1.1 (Lohse & Weir, 2021; GenBank accession GCA_905333055.1, RefSeq GCA_905333055.1) using STAR version STAR_2.4.0g1 (Dobin et al., 2013). Gene expression quantification was carried out using StringTie (Pertea et al., 2015) (Tables S2 and S3). The proteome annotation used to identify genes is available from the European Bioinformatics Institute through https://ftp.ebi.ac.uk/pub/ensemblorganisms/Maniola_jurtina/GCA_905333055.1/ensembl/geneset/2021_06/ (protein‐coding genes pep.fa.gz). Differential gene expression analysis was conducted using the DESeq2 (version 1.38.3) (Love et al., 2014) package in RStudio accounting for batch effects within the data (Tables S4–S9). The dataset was filtered to only include genes which had a base mean >5, log_2_ fold change >1 and adjusted *p*‐values <0.05 to reduce the inflation effect of noise at low expression levels. The base mean is defined as the average of the normalised FPKM count values, divided by size factors, taken over all samples. Each of the four tissues were compared to the other tissues to identify genes significantly differentially expressed (upregulated or downregulated). Plots were created using RStudio packages as follows: heatmaps of the top 50 genes with the highest and top lowest fold change were generated using ComplexHeatmap 2.14.0 (Gu, 2022); Volcano plots and principal component analysis (PCA) plot were generated using ggplot 3.4.4 (Wickham, 2016); the Venn diagram was created using venneuler 1.1‐4 (Gao et al., 2021). Expression analysis of candidate sensory genes used FPKM values averaged across replicates; these were then normalised as *z*‐scores to visualise subtle but consistent gene expression differences.

### Annotation of sensory‐related genes

Genes classified as chemosensory or phototransduction genes were extracted from the *M. jurtina* genome using two different approaches. For chemosensory‐related genes, functional domains were annotated in all predicted proteins using PfamScan (command line tool pfam_scan.pl) to search against the Pfam‐A.hmm database with cutoff –cut_ga and an e‐value threshold of 1e‐3 (Mistry et al., 2021). Chemosensory proteins were extracted by searching for Pfam domains associated with each gene subtype (odorant receptor; 7tm_4, gustatory receptor; 7tm_7, ionotropic receptor; Lig_chan, odorant binding protein; PBP_GOBP, chemosensory binding protein; OS‐D, sensory neuron membrane protein; CD36). Genes involved in the phototransduction pathway, including the opsin genes, were annotated using BLASTp of a seed dataset of protein sequences obtained from Ernst and Westerman (2021), Macias‐Muñoz et al. (2019), and Mulhair et al. (2023) against the *M. jurtina* predicted proteome.

### Discovery of novel genes

Predicted proteome data from 50 species (17 Nymphalidae, 32 other Lepidoptera, 1 Trichoptera) were obtained from Ensembl Rapid Release http://rapid.ensembl.org (accessed August 2024); of these, 42 were generated by the Darwin Tree of Life Project (BioProject PRJEB40665; Darwin Tree of Life Consortium, 2022). Taxon sampling was designed to achieve robust phylogenetic coverage across Lepidoptera while also preferentially selecting species with proteome predictions‐based RNA sequence data. Gene annotations were filtered to retain the longest transcript for each gene and OrthoFinder v2.3.14 was run with default parameters to determine orthogroups within the dataset (Emms & Kelly, 2019). To relate these to a species tree, amino acid sequences from 1528 single copy orthologues from the OrthoFinder output and present in all species, were aligned using MAFFT v7.505 (Katoh & Standley, 2013), trimmed using trimAl v1.4.rev15 build (Capella‐Gutiérrez et al., 2009), and all alignments were then concatenated with PhyKIT (Steenwyk et al., 2021). The concatenated alignment was used to generate a species tree using IQ‐TREE version 2.0‐rc1 (Minh et al., 2020) using 1000 bootstrap iterations, the LG + G4 model and option ‐nt AUTO which automatically determines the best number of cores given the current data and computer capacity. Orthogroups gained on the branch leading to the Nymphalidae were extracted using Orthoparser (github.com/PeterMulhair/ortho_parser). Genes within orthogroups were analysed to generate expression matrices, explore gene copy number and conduct synteny analyses. Figures including phylogenetic trees and heatmaps generated in R used ggtree (Yu et al., 2017), ggplot2 (Wickham, 2016), and Pheatmap (Kolde, 2025) and were edited using Affinity Designer 2 (Serif, 2024).

## RESULTS

We hypothesised that any divergent transcriptomic profiles underpinning the difference observed in nymphalid T1 legs may result from either novel genes arising on the node leading to Nymphalidae or from shifts in expression of pre‐existing genes due to co‐option of new regulatory networks. To investigate this we divided our analyses into three main parts: (i) a comparison of T1 to T2 and T3 legs, (ii) a comparison of T1 to palps and (iii) a detailed investigation into the molecular basis of expression similarities arising between T1 and palps. We also assessed the contribution of novel genes versus existing genes which have gained distinct expression profiles coincident with the evolution of the reduced T1 legs.

### Transcriptomic differences between T1 legs and walking legs

**FIGURE 1:**
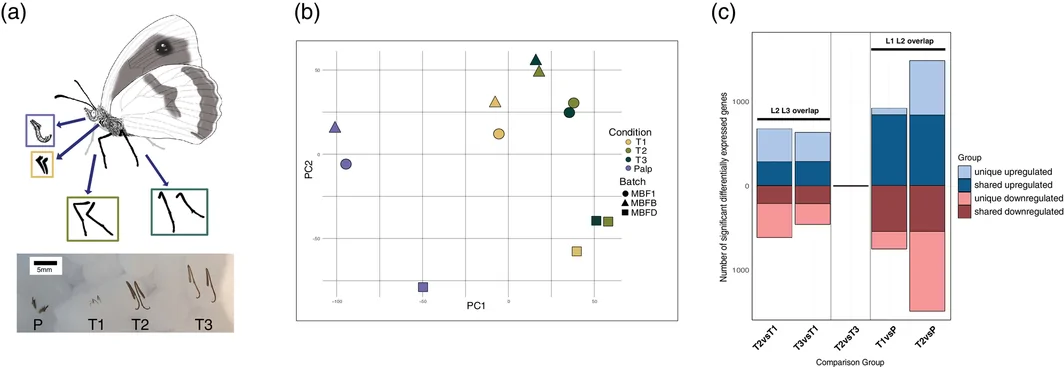
(a) *Maniola jurtina* illustration demonstrating dissection of palps and T1, T2 and T3 legs. Scale bar 5 mm. (b) Principal component analysis (PCA) analysis of transcriptomes from T1, T2 and T3 legs, and palps. PC1 and PC2 explain 46% and 29% of the variance respectively. (c) Number of genes significantly ‘upregulated’ (higher expression) and ‘downregulated’ (lower expression) between, from left to right: T2 versus T1, T3 versus T1, T2 versus T3, T1 versus palps, T2 versus palps. The darker shading shows the number of upregulated and downregulated genes in common between T2 versus T1 and T3 versus T1 (left hand bars), or in common between T1 versus palps and T2 versus palps (right hand bars). Note that gene expression is essentially identical between T2 and T3, that gene expression is T1 legs is different to T2 and T3, T1 gene expression is more similar to palps than T2 gene expression is to palps, and T1 gene expression is more similar to other legs than it is to palps.

To obtain a dataset suitable for conducting a differential gene expression analysis, *M. jurtina* butterflies were collected, dissected to remove palps, T1, T2 and T3 legs respectively (Figure 1a), and pooled to produce 3 replicates consisting of 6 or 15 females, with 15 males pooled to form the single male sample described in Table S1. We focused on samples consisting of female *M. jurtina* specimens for differential gene expression analyses, conducted using DESeq2 with the percentage of uniquely mapped reads ranging between 50% and 74% across samples. Gene expression quantification was measured with the gene models of *M. jurtina* using StringTie (Pertea et al., 2015; Tables S2 and S3). First, to determine whether there were broad expression profile differences between tissues, we constructed a PCA plot from these gene expression quantifications of palps, T1, T2 and T3 legs. Despite a consistent batch effect visualised along PC2, possibly related to collection date, PC1 demonstrated larger variance (46% vs. 26%) pointing to tissue type as the main driver of gene expression difference. The analysis reveals that palp gene expression has the greatest dissimilarity to the other tissue types, while T2 and T3 legs have the smallest difference between them, indicated by the close grouping of these data points (Figure 1b). T1 legs do not cluster with other tissues but do show more apparent similarity to T2 and T3 legs than to palps. This suggests that T1 legs have a gene expression profile which is not typically found in palps or in the other legs (Figure 1b).

To quantify the differences in gene expression between tissues, pairwise comparisons were conducted across all four tissue types (P, T1, T2 and T3), evaluating each dataset against the others in a like‐for‐like manner (e.g., T1 vs. T2 etc.; Tables S4–S9). Differentially expressed genes underwent filtering to only include genes which had a base mean >5, log_2_ fold change >1 and adjusted *p*‐values <0.05. When comparing T2 walking legs against T3 walking legs, only one gene was found to be differentially expressed and has putatively lower expression in T2 in comparison to T3 (ENSMJUG00000000611, an olfactory receptor) (Figure 1c). This indicates there is little, transcriptomic difference between T2 and T3 legs. We therefore used T2 as a representative of the walking legs in further analyses. When comparing the reduced legs of T1 versus the walking legs of T2, however, 791 differentially expressed genes were identified (Figure 1c). Of these 402 showed higher expression in T1 and 389 had lower expression in T1. This indicates a clear transcriptomic difference in the reduced legs compared with the walking legs.

### Larger differences in expression profile observed between legs and palps

In a comparison of T1 legs versus palps, we found 1718 differentially expressed genes. Of these, 972 showed higher expression in T1 and 746 had lower expression in T1. In a comparison of palps versus T2 legs, we found 2864 differentially expressed genes. Of these, 1380 had higher expression in the palps and 1484 had lower expression in the palps. This suggests that palps have a very different transcriptomic profile to legs, but the difference is greatest when compared to walking legs (Figure 1c). In addition, although the reduced legs of T1 differ in gene expression to the walking legs (T2 and T3), all legs share more similar transcriptome profiles to each other than to palps (Figure 1b).

To investigate the nature of the gene expression differences between the reduced legs (T1) and walking legs (T2, T3), we first searched for gene expression differences unique to T1 legs. Of the genes differentially expressed between T1 versus palps and T1 versus T2, 106 genes were shared across both analyses (Figure S1, Table S10). These represent gene expression differences specific to T1 legs as compared to palps, T2 legs or T3 legs. Of these, 47 were more highly expressed in T1 and 59 were expressed at a lower level in T1 in both analyses. The 47 loci upregulated in T1 legs include a gene encoding a putative mechanosensory ion channel homologous to the *nanchung* gene from *Drosophila* (ENSMJUG00000008717), two putative serotonin receptor genes (ENSMJUG00000001776, ENSMJUG00000008930), the blue‐sensitive opsin gene (ENSMJUG00000015046) and three conserved transcription factors (vnd homeobox gene ENSMJUG00000015716; unpg/Gbx homeobox gene ENSMJUG00000001782; a forkhead domain gene ENSMJUG00000013797). This indicates that T1 legs may have acquired sensory and biochemical functions different to either of the other analysed tissues.

### Nymphalid reduced legs have acquired aspects of palp‐like gene expression

**FIGURE 2:**
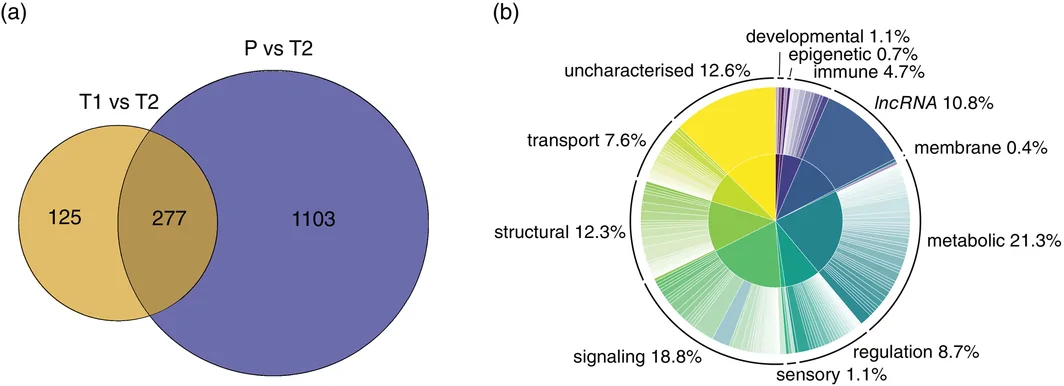
Genes of interest explored from the genes upregulated in both T1 legs and palps: (a) common upregulated genes between T1 and palps showing a crossover of 277 genes common and upregulated in both T1 legs and palps. (b) Functional category (solid colour, inner pie chart) and in‐depth functions (differentially coloured, outer pie chart) of the 277 genes which were upregulated in both T1 legs and palps. See Figure S2 for details on the relationship between pie chart colour and functional category breakdown.

Although there are large gene expression differences between palps and legs, it is possible that the reduced legs of Nymphalidae acquired some palp‐like characters. To test for this, we searched for genes upregulated or downregulated in palps (compared to T2 legs) that are also upregulated or downregulated in T1 (compared to T2 legs). We found 277 genes in the upregulated category: ‘palp’‐enriched genes also enriched in expression in T1 legs (Figure 2a; Table S11). We found 286 genes in the downregulated category: these genes have significantly lower expression in palps and T1 legs than in T2 legs (Table S12). These include several genes encoding putative transcription factors, for example, *Antp* and *Ubx* genes likely reflecting the spatial position of the appendages, *Lbx* homeobox gene, *Pax1/9* (*Pox‐meso*), *Pax3/7* and two T‐box genes. A control analysis searching for genes upregulated in both palps and T2 legs, compared to T1 legs as a common reference, gave a lower number: 59 genes (Table S13).

The large number of ‘palp‐ and T1‐upregulated genes’ and ‘palp‐ and T1‐downregulated genes’ suggests that T1 legs may have acquired some of the biochemical functions typically associated with palps. In both analyses, we do not exclude genes that are differentially expressed between palps and T1 legs, as we aim to detect limited transcriptomic similarities as well as large scale convergence. For example, of the 277 of ‘palp‐ and T1‐enriched genes’, 25 are differentially expressed between palps and T1 legs (Table S11). To delve deeper into the *M. jurtina* genes identified, Pfam annotation and a BLASTp search were conducted against the *D. melanogaster* predicted proteome (Table S11) to examine their putative functions. A nested pie chart was generated to visualise the overall functional category, combined with specific function for each of the genes identified in the 277 T1/palp upregulated genes (Figure 2b). The majority of genes were classed as metabolic (21.3%), while a large proportion of genes were uncharacterised or lncRNA (13% and 11% respectively). Genes in the sensory and developmental function categories each comprised only 1% of these genes (Figure 2b, Table S11); these include a putative chemosensory protein (ENSMJUG00000005750) and a POU class homeobox gene (ENSMJUG00000013017).

**FIGURE 3:**
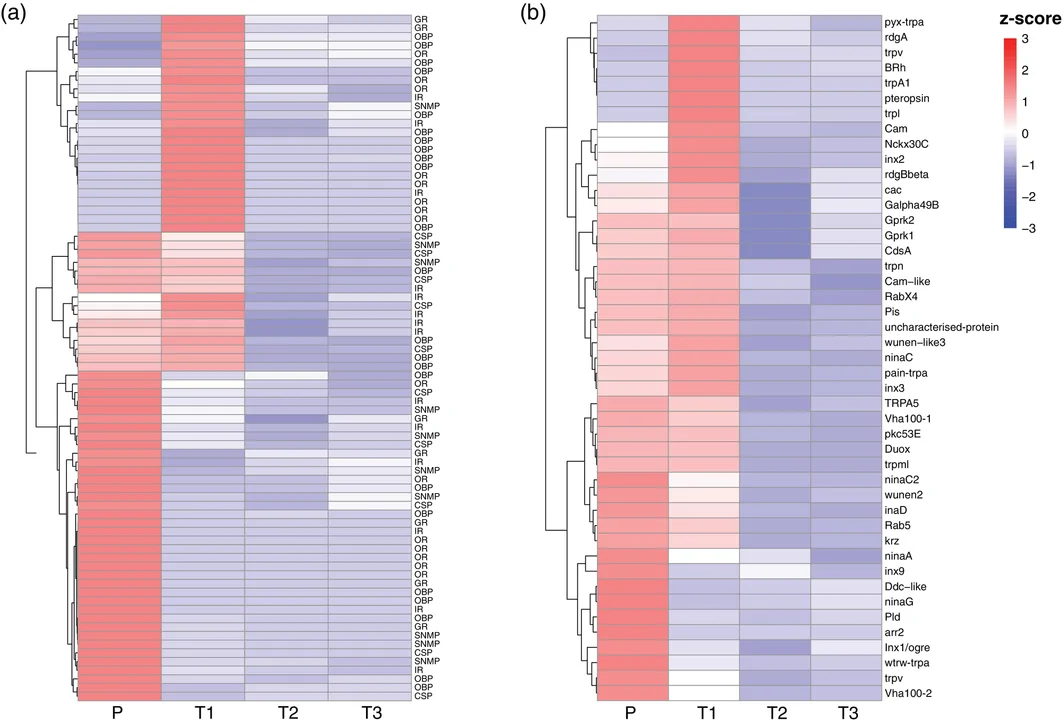
*z*‐Score heatmaps showing upregulation of (a) chemosensory and (b) phototransduction genes in palps (P), T1 legs (T1) or both tissues. Higher *z*‐scores (red) represent higher rates of gene expression relative to T2 and T3 legs. Gene type (chemosensory subtype) or gene name (phototransduction genes) are shown on the right of each expression row.

As a complementary approach, we also examined the expression of predicted sensory genes in the RNAseq datasets to ensure physiologically relevant genes which did not meet the statistical threshold for differential expression were not overlooked. Specifically, we examined genes encoding proteins involved in chemosensation, notably olfactory binding proteins (OBP), chemosensory proteins (CSP), sensory neuron membrane proteins (SNMP), ionotropic receptors (IR), gustatory receptors (GR) and olfactory receptors (OR), as well as genes involved in the phototransduction pathway, which include the visual opsins. For each gene family, we identified homologues in the *M. jurtina* genome and calculated FPKM values of gene expression in palps, T1, T2 and T3 legs. Expression values were averaged across female replicates for all tissues (Figures S3A and S4A; Tables S14 and S15), and *z*‐score was calculated to allow for comparison across tissues for each gene (Figure 3, Figures S3B and S4B). In total, we extracted 182 chemosensory‐related genes, consisting of 60 ORs, 28 GRs, 32 IRs, 29 OBPs, 18 CSPs and 15 SNMPs and 65 phototransduction genes, including the 3 primary visual opsin genes (UV, Blue and longwave‐sensitive opsins), from the *M. jurtina* genome. Of the chemosensory genes analysed, 129/182 had at least some level of expression (FPKM > 0) in one of the tissues. We found 37 genes with a positive *z*‐score in palps suggesting they may have a specialised sensory function in palps relative to legs. We also found 25 chemosensory‐related genes with a positive *z*‐score only in T1 legs (Figure 3a); these could represent sensory functions unique to the reduced legs, at least compared to palps and the other legs. Finally, we found 17 chemosensory‐related genes with a positive *z*‐score in both palps and T1 legs, suggestive of a shared sensory function. Overall, palps and T1 legs displayed a higher number of chemosensory genes with unique or shared expression (79 genes), as deduced from positive *z*‐scores, when compared to T2 or T3 legs (19 genes; Figure S3B). Of the 66 phototransduction genes, 63 had some evidence of expression in one of the four tissues (Figure S4A). Of these, 23 showed overlap in P and T1 relative to T2 and T3, 7 genes showed more specific expression in T1, and 15 had palp‐specific expression (Figure 3b, Figure S4B). Interestingly, of the visual opsin genes, the blue‐sensitive opsin (BRh) was the only one with evidence of expression and showed expression specific to T1 legs. BRh showed a higher level of expression in the T1 legs (average FPKM across replicates of 6.7) versus the other tissues (palps 0.23; T2 legs 0.39; T3 legs 0.2). Other genes showing diverged T1 leg expression and with central roles in visual pathways include retinal degeneration A (palps 7.06; T1 legs 10.38; T2 legs 7.45; T3 legs 7.10) that encodes a diacylglycerol kinase, which is required for the fast and sensitive response of photoreceptor cells.

### A nymphalid‐specific gene duplication upregulated in palps and T1 legs

**FIGURE 4:**
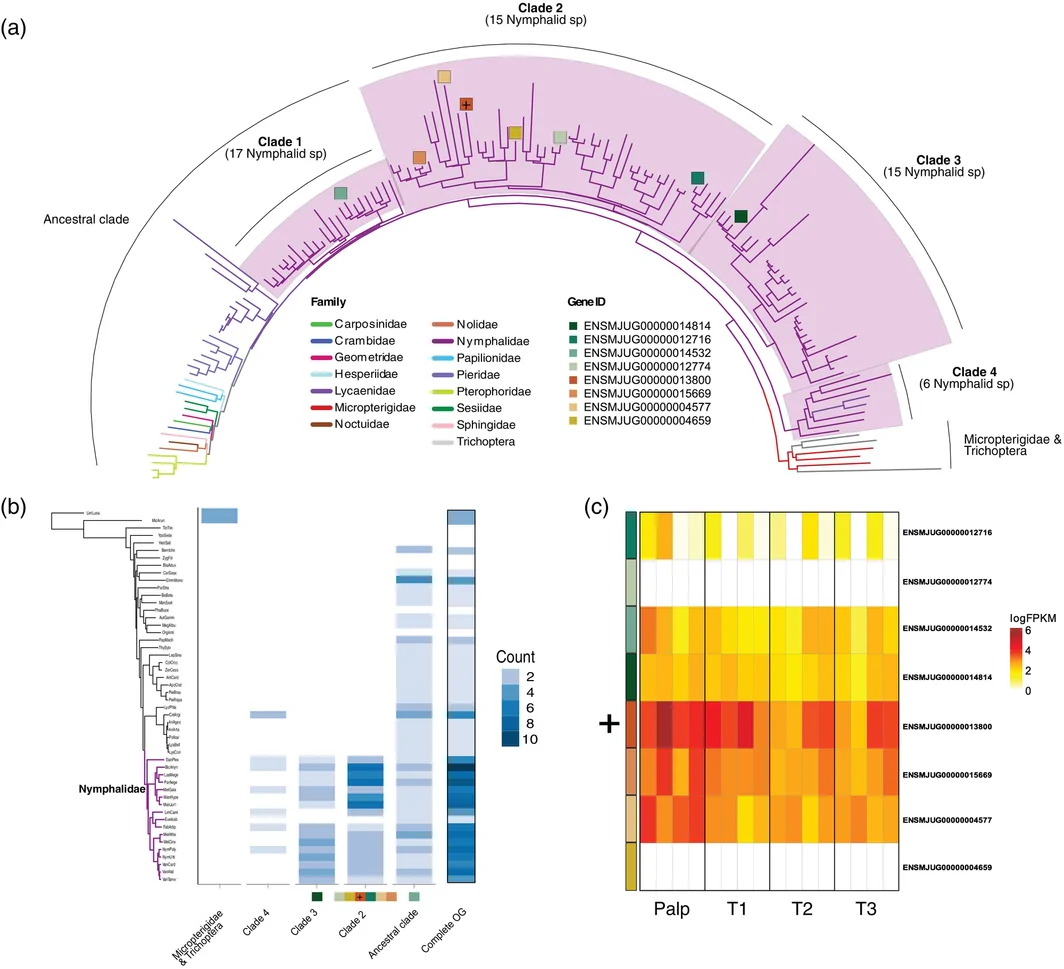
In‐depth analysis of a nymphalid‐specific gene duplication generating a gene upregulated in both T1 legs and palps. (a) Phylogenetic tree (coloured by lepidopteran family) of orthogroup OG0000217 containing putative Nymphalidae‐specific gene duplications. The specific gene copy which is upregulated in *Maniola jurtina* T1 legs and palps is denoted by +. The number of unique nymphalid species per clade is noted; numbers per species are given in Table S18. (b) Gene count for each species used in this analysis in the orthogroups of interest, split into clades with overall gene copy in the orthogroup on the right‐hand side. Species names and gene copy number in Table S18. (c) LogFPKM expression across palps, T1, T2 and T3 legs for each of the *M. jurtina* genes in these orthogroups of interest. The gene which is upregulated in T1 legs and palps is denoted by +. Only three of these gene copies demonstrate expression in any of palps, T1, T2 or T3.

For each of the 277 genes identified as upregulated in both T1 legs and palps (compared to T2 legs), we tested if these were members of multigene families and whether copy number in these gene families had changed during the evolution of the Nymphalidae, using genomic data from 17 Nymphalidae, 32 other Lepidoptera and a Trichoptera outgroup (Table S16, Figure S5). The 277 genes of interest included 246 putative protein‐coding genes (the remaining are annotated as non‐coding genes), belonging to 235 gene families (‘orthogroups’). Of these, 8 orthogroups had evidence of recent gene duplication or were only identified in the Nymphalidae subfamily Satyrinae plus *Danaus plexippus*, that is, not present in any species outside this clade. One orthogroup was notable as present, typically in single copy, in nearly all species used in this analysis (all Lepidoptera species and Trichoptera outgroup), but with clear patterns of nymphalid‐specific gene duplication events (OG0000217) (Figure 4a,b). The lowest gene copy number in the Nymphalidae is observed in *Eueides isabella* (1 copy) with the highest found in *Bicyclus anynana* (11 copies) and a mean of 6 gene copies across all nymphalid species analysed here (Table S17). Phylogenetic analysis suggests that within the Nymphalidae, duplications of this gene generated four clades (gene subfamilies), with some subfamilies undergoing further lineage‐specific duplication (Figure 4a; Table S18).

Although eight *M. jurtina* genes were identified within this orthogroup, only one of these genes was present in the upregulated group of 277 T1/palp‐expressed genes (ENSMJUG00000013800) (Figure 4c). Six genes were expressed in some or all of the tissues investigated (ENSMJUG00000012716, ENSMJUG00000014532, ENSMJUG00000014814, ENSMJUG00000013800, ENSMJUG00000015669 and ENSMJUG00000004577). Two gene copies have higher expression in the palps (ENSMJUG00000015669, P vs. T2 padj = 0.029 and ENSMJUG00000004577, P vs. T2 padj = 0.0001), which had an average FPKM value of 35 and 38 respectively in palps, compared to 21 and 17 respectively across all leg tissues, while the remaining three genes had relatively consistent expression patterns across all four tissue types (Figure 4c). This suggests the paralogous gene copies have diverged in their expression domains. For all *M. jurtina* sequences in this orthogroup, a BLASTp search against *D. melanogaster* resulted in hits to uncharacterised proteins, with the top hit for ENSMJUG00000013800 being CAL85485 (gene ID: CG9649) with a percentage identity of 29.930 and an e‐value of 8.69e^−27^. All proteins were annotated as possessing a trypsin domain (Pfam domain PF00089.29), placing these genes as part of the S1 family of peptidases (Rawlings & Barrett, 1994).

### Novel genes do not underpin transcriptomic differences in T1 legs

**FIGURE 5:**
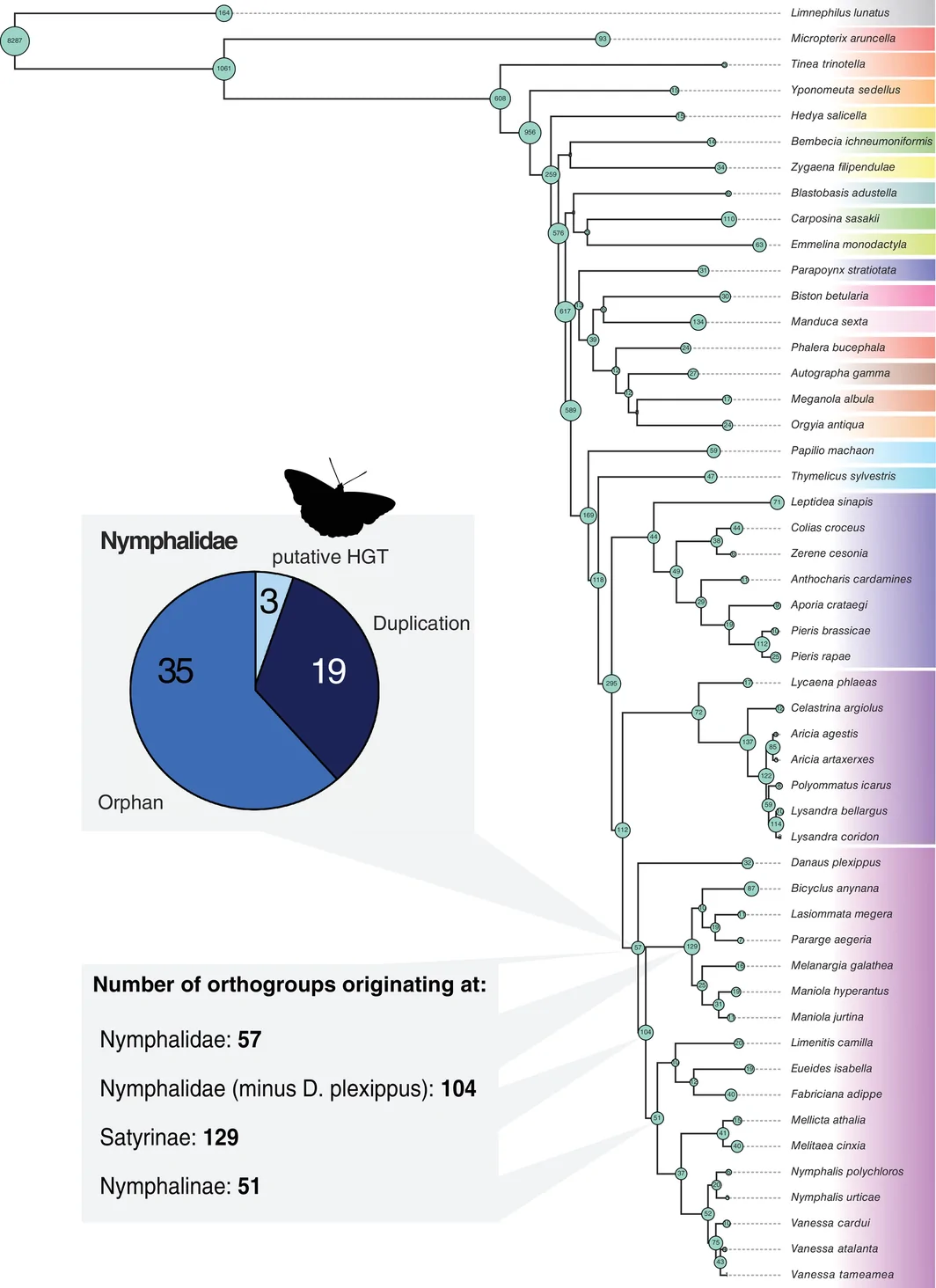
Genes emerging on the Nymphalidae butterfly node. Species tree showing the number of orthogroups gained at each phylogenetic node. Lepidopteran families are labelled by coloured boxes. Pie charts show the number of orthogroups originating at the Nymphalidae, Nymphalidae minus *Danaus plexippus*, Satyrinae and Nymphalinae nodes and proportions of the putative modes of new gene origin.

To investigate whether any transcriptomic diversity observed in the T1 legs arose from novel genes arising on the node leading to the Nymphalidae, we first built a phylogenetic tree using 1528 single copy genes from across the same 50 species as used above, onto which gene gain could be mapped. The species selected were representative of 21 lepidopteran families and one non‐lepidopteran insect (*Limnephilus lunatus*, order Trichoptera) to give strong taxonomic coverage across Lepidoptera. The analyses above included some examples of pre‐existing genes in Lepidoptera (trypsin peptidases and chemosensory genes) showing patterns of duplication and/or differential gene expression in the palps and T1 legs of *M. jurtina*. To assess whether additional novel genes or novel gene families emerged in Nymphalidae we first constructed homologous gene groups (‘orthogroups’) from all 50 species in our dataset using OrthoFinder (Emms & Kelly, 2019). Novel gene families here are defined as orthogroups present in a clade but absent from all outgroup taxa, that is, taxonomically restricted genes (Hoile et al., 2025). We identified 57 gene families originating on the branch leading to the Nymphalidae (Figure 5, Figure S6). Interestingly, even larger numbers of gene gain events are observed on nodes subsequent to the emergence of Nymphalidae: 104 genes originating on the node containing non‐Danainae nymphalids and 129 genes on the branch leading to the subfamily Satyrinae.

Of the 57 Nymphalidae‐specific orthogroups, we sought to classify their putative mode of origin. To do so we carried out BLASTp searches against the nr database filtered by (i) removing all lepidopteran proteins and (ii) removing all metazoan proteins. Those with hits to dataset (i) were classified as duplicated from ancestral gene families, dataset (ii) as putative horizontally transferred genes, and those with no BLAST hits as orphan genes. We deduce that 19 orthogroups likely arose via duplication followed by extensive sequence divergence (Table S19). Putative HGTs account for only three orthogroups, as indicated by presence in non‐metazoan genomes but absent from animals other than nymphalid butterflies. We suggest that the majority, 35 orthogroups, are orphan genes, genes which have either emerged by de novo gene genesis or have diverged in their amino acid content to such an extent as to not be detectable by standard sequence identity searches. None of the 57 genes were determined as differentially expressed in the palps or T1 legs compared to T2 and T3 legs. Indeed, only 7 of these orthogroups possessed genes in *M. jurtina* which had any level of expression in palps or legs. This suggests that while new genes did arise during the evolution of Nymphalidae, it is unlikely they made a major contribution to the novel biology of nymphalid T1 legs.

## DISCUSSION

Previous research has established serial homology between legs and labial palps, with evidence from similar developmental gene expression and homeotic transformations. This serial homology extends to the legs of the T1 segment (Hughes & Kaufman, 2002; Panganiban et al., 1994). However, serial homologues can diverge in structure and function in evolution, presumably underpinned by gene expression differences (Monteiro, 2021; Monteiro et al., 2025). While there has been research assessing differences in gene expression between sensory tissues, including legs, in butterflies (Briscoe et al., 2013; van Schooten et al., 2020; Wu et al., 2022), we know of no prior research that has examined the transcriptomic similarities and differences between the highly diverged T1 legs of nymphalid butterflies and their serial homologues. Here we have examined these differences in adult tissues of a nymphalid butterfly, although we stress that additional genes may have been active during development of these serially homologous appendages.

We first established that there are few transcriptomic differences between T2 and T3 adult legs. Only one gene was identified as differentially expressed between T2 and T3 legs, emphasising a high degree of transcriptomic similarity, at least in females. Similarity was also observed in the PCA analysis, demonstrating a low proportion of variance between T2 and T3 (Figure 1b). Although a higher proportion of variance was observed between T1 and walking legs (T2 and T3), PCA analysis concluded that T1 legs have a higher degree of transcriptomic similarity to walking legs than they do to palps (Figure 1b). This is also supported by a greater number of differentially expressed genes observed between T1 versus palps in comparison to T1 versus T2 (Figure 1c). This confirms the hypothesis that T1 legs have transcriptomic similarity to walking legs (T2 and T3) (Figure 1c).

Although T1 legs share a closer gene expression profile to the walking legs than they do to palps, they have still acquired some gene expression similar to that of the palps distinct from that found in walking legs (Figure 1b,c). We found 277 genes significantly upregulated in both T1 legs and palps, and 286 genes significantly downregulated in T1 legs and palps, indicating that T1 may have acquired some of the expression and, by consequence, function typically associated with the palps. Additionally, T1 legs have some genes which are not upregulated in any of the palps, T2 or T3 legs (Figure 1c and Table S10). We also identified 124 sensory‐related genes expressed in either palps, T1 legs or both tissues when compared to T2 and T3 legs, although not all of these were identified in the differential gene expression analysis (Figure 3). This suggests that, along with diverging in their expression profiles where they are closer to palps than the walking legs are, T1 legs may have also acquired expression and potential functions not found in either of these three tissues; exploring the specific function of these in the context of T1 leg biology or physiology will be an interesting area to explore in future research.

Having established that T1 legs have acquired some transcriptomic similarity to palps, we were interested to know if some of the transcriptomic differences characterising reduced T1 legs could be related to evolution of new genes or expanded gene families. From the genes which were found to be upregulated in T1 and palps, when compared to walking legs, eight orthogroups to which some of these genes belong were present in *D. plexippus* and the Nymphalidae subfamily Satyrinae only, within our analysed data set. Interestingly, the Satyrinae have the highest number of novel genes emerging on their node of origin (129) in comparison to the subfamily Nymphalinae (51) and the Nymphalidae family itself (57) (Figure 5). Research suggests that Satyrinae often exhibit greater host plant specialisation in comparison to other subfamilies within Nymphalidae, for example, Nymphalinae (Nylin et al., 2014). An example of this is many Satyrinae predominantly utilising plants from the order Poales (grasses), while Nymphalinae typically have a broader range of host plants across multiple plant orders. As such, it is possible that the orthogroups which only contain Satyrinae and Danainae genes assist in this greater host plant specificity (Nylin et al., 2014). One orthogroup was identified as having genes present in almost every species used in the study (average of one gene copy per non‐Nymphalidae species), with a marked increase in copy number occurring in 16 out of 17 Nymphalid species in our dataset (OG0000217) (Figure 4a,b). We annotated this gene family as consisting of trypsin‐related proteins based on their functional domain content. Trypsins are a family of serine protease enzymes which play a role in protein digestion by breaking down polypeptide chains into smaller peptides and amino acids. They are typically involved in digestion but may have other roles (Brenner, 1988; Rawlings & Barrett, 1994). Literature investigating trypsin‐like gene expression in any arthropod legs is limited, however, several serine proteases and protease inhibitors have been identified in the crustacean olfactory organ, and it suggested that they may play a role in perireception (e.g., odour activation or inactivation) or in the development or survival of olfactory receptor neurons (Johns et al., 2004). The diverse expression pattern observed for *M. jurtina* genes in this orthogroup suggests that duplication and divergence may have taken place within this orthogroup and therefore the genes may have adopted new functions in nymphalid butterflies. It is possible that these trypsin domain‐containing genes may play a role in sensory perception in Nymphalidae T1 legs and palps, however further research is required to test this hypothesis.

As palps are known to have some sensory function, and we found that T1 legs have some transcriptomic similarity to palps, we also explored the potential role of sensory genes in nymphalid T1 legs. We found 25 chemosensory genes showing highest expression in T1 legs compared to palps and walking legs, with the majority of these being olfactory binding proteins (11/25) and olfactory receptors (8/25; Figure 3a). Previous research has identified gustatory and olfactory receptors as mediators of insect‐plant reactions through identification of secondary plant compounds as deterrents or attractants of insect oviposition and feeding; however, the exact role of reduced T1 legs in this is not yet clear (Baur et al., 1998; Calvert & Hanson, 1983; Fox, 1966; Renwick & Chew, 1994; Wolfe et al., 2011). It is possible, however, that specialised receptors on the T1 legs, which may be unique among specific Nymphalidae species, may allow for host‐specific interactions with certain plants (Briscoe et al., 2013). Examining genes involved in the phototransduction cascade also revealed extraocular expression of blue opsin in T1 legs. Non‐visual and extraocular opsin expression has been observed in *D. melanogaster*, crustaceans, cephalopods and fish (Kingston & Cronin, 2016). Retinal non‐visual receptors and associated opsins have been identified across animal tissues and are thought to utilise the phototransduction pathway to detect light for non‐visual purposes (Feuda et al., 2022; Kingston & Cronin, 2016). Extraocular colour sensing has also been observed in *Biston betularia* (Peppered moth) caterpillars, allowing them to choose to rest on colour‐matching twigs for camouflage, even when ocelli are obscured (Eacock et al., 2019). Another role for extraocular vision involves regulation of circadian clocks. Entrainment of the circadian rhythms throughout the insect body via opsin expression can provide important cues for complex behaviours such as sleep, mating and migration (Leung & Montell, 2017; Merlin et al., 2009). Beyond this, opsins have been shown to be involved in thermosensation (Shen et al., 2011), taste sensation (Leung et al., 2020) and even hearing (Senthilan et al., 2012) in *D. melanogaster*. Our findings suggest that the reduced T1 legs have acquired some sensory function which is not observed in walking legs in *M. jurtina*.

We therefore conclude that T1 legs in Nymphalidae butterflies have sensory function and that some transcriptomic similarity is observed between T1 legs and palps. However, it is notable that T1 legs still bear more gene expression similarity to walking legs than they do to palps.

## AUTHOR CONTRIBUTIONS

**Asia E. Hoile:** Conceptualization; investigation; writing – original draft; methodology; visualization; writing – review and editing; formal analysis; data curation; resources. **Peter O. Mulhair:** Conceptualization; investigation; methodology; visualization; writing – review and editing; formal analysis; data curation; supervision. **Peter W. H. Holland:** Conceptualization; writing – original draft; methodology; writing – review and editing; project administration; supervision; formal analysis; funding acquisition.

## CONFLICT OF INTEREST STATEMENT

The authors declare no conflicts of interest.

## Supporting information


**Figure S1.** Gene expression unique to T1 legs. (A) Upregulated and (B) downregulated genes from differential gene expression analyses comparing T1 legs versus Palps (blue) and T1 legs versus T2 legs (yellow). Genes which are upregulated and downregulated in T1 in both comparisons (47 and 59 respectively) are shown in the overlap.
**Figure S2.** Functional categories Specialised functions of different categories of genes observed as upregulated and differentially expressed in both T1 and Palp when compared with T2, as displayed in Figure 2.
**Figure S3.** Chemosensory gene expression in all tissues. Expression values given as FPKM in log_10_ (A) and *z*‐scores (B) for all annotated chemosensory genes in Palp, T1, T2, and T3 legs. Gene identification numbers and their corresponding gene type are given at the end of each row.
**Figure S4.** Phototransduction gene expression in all tissues. Expression values given as FPKM in log_10_ (A) and *z*‐scores (B) for all annotated phototransduction genes in Palp, T1, T2, and T3 legs. Gene identification numbers and their corresponding gene name are given at the end of each row.
**Figure S5.** Gene copy number of 277 palp and T1 enriched genes. Species tree of lepidopteran species used in this study. Heatmap to the right corresponds to the copy number of the orthogroups ordered by number of gene copies present from left to right. These orthogroups contain genes in *Maniola jurtina* which are upregulated in palps and T1 legs relative to T2 legs.
**Figure S6.** Gene gain across Lepidoptera phylogeny. Species tree of Lepidoptera used in this study with nodes showing the number of orthogroups gained on each branch (as shown by number and scaled green circles). Bar chart of the right shows the number of orthogroups containing each corresponding species.

**Table S1.** Specimen collection data, showing which individuals were pooled for RNA sequencing and the corresponding NCBI SRA accession numbers.
**Table S2.** Raw reads counts per sample for all loci. Sample names along the top: T1 legs, T2 legs, T3 legs, Labial palps for each replicate.
**Table S3.** fpkm per sample for all loci.
**Table S4.** DESeq2 output for the comparison T1 legs versus T2 legs giving baseMean, log_2_FoldChange and statistical analysis. Positive log_2_FoldChange values indicate higher expression in T1 compared to T2.
**Table S5.** DESeq2 output for the comparison T1 legs versus T3 legs giving baseMean, log_2_FoldChange and statistical analysis. Positive log_2_FoldChange values indicate higher expression in T1 compared to T3.
**Table S6.** DESeq2 output for the comparison T2 legs versus T3 legs giving baseMean, log_2_FoldChange and statistical analysis. Positive log_2_FoldChange values indicate higher expression in T2 compared to T3.
**Table S7.** DESeq2 output for the comparison T1 legs versus Palps legs giving baseMean, log_2_FoldChange and statistical analysis. Positive log_2_FoldChange values indicate higher expression in T1 legs compared to palps.
**Table S8.** DESeq2 output for the comparison T2 legs versus palps legs giving baseMean, log_2_FoldChange and statistical analysis. Positive log_2_FoldChange values indicate higher expression in T2 legs compared to palps.
**Table S9.** DESeq2 output for the comparison Palps versus T3 legs giving baseMean, log_2_FoldChange and statistical analysis. Positive log_2_FoldChange values indicate higher expression in Palps compared to T3 legs.
**Table S10.** Identification of T1‐enriched genes. Column A gives loci enriched in T1 legs compared to Palps. Column B gives loci enriched in T1 legs compared to T2. These two lists are then compared to give two indications of T1‐specific features (within this dataset), as follows. Column F gives the 47 loci which are more highly expressed in T1 legs in both comparisons (higher in T1 than palp, and higher in T1 than T2). Column G gives the 59 loci that are expressed less in T1 legs in both comparisons (lower in T1 than palp, lower in T1 than T2). Columns F and G combined gives 106 loci.
**Table S11.** List of 277 loci enriched in Palp and T1 legs compared versus T2 legs, giving gene ID, orthogroup, top BLAST hit, putative gene type based on BLASTp, Pfam ID, and mean fpkm per tissue type. The 25 genes shaded in pink in column 1 are DE (differentially expressed) between Palp and T1 legs.
**Table S12.** List of 286 loci with lower expression in Palp and T1 legs compared versus T2 legs.
**Table S13.** List of 59 loci enriched in Palp and T2 legs compared versus T1 legs.
**Table S14.** Putative chemosensory loci: fpkm for each individual sample.
**Table S15.** Putative phototransduction genes: fpkm for each sample.
**Table S16.** The 50 proteomes used in the analysis of gene origins and gene family expansions in Nymphalidae evolution, giving accession numbers, project provider and references.
**Table S17.** Gene count within OG0000217 for each species.
**Table S18.** Gene count within each subclade of OG0000217 for each species.
**Table S19.** Information on mode of origin, BLAST hits, and pfam functional domain annotation for 57 OGs gained on the Nymphalidae branch.

## Data Availability

RNAseq data generated in this study are deposited under NCBI BioProject PRJNA1303389 (Hoile et al., 2026a; https://www.ncbi.nlm.nih.gov/bioproject/PRJNA1303389/). Code required to reproduce results is deposited on Figshare (Hoile et al., 2026b; https://doi.org/10.6084/m9.figshare.29804459). Supplementary tables and Supplementary figures referred to in the manuscript are deposited on the ORA‐Data Research Archive (Hoile et al., 2026c; https://doi.org/10.5287/ora-e9jxdvpk6).
