# Supplementary material for: Distinctive gene expression in the reduced first thoracic legs of a nymphalid butterfly
Source: Insect Mol Biol. 2026 Jun 2;35(5):504–16. doi: 10.1111/imb.70045 (PMC13551524; doi:10.1111/imb.70045)
Supplement: Supplementary file 1 [file IMB-35-504-s001.pdf]

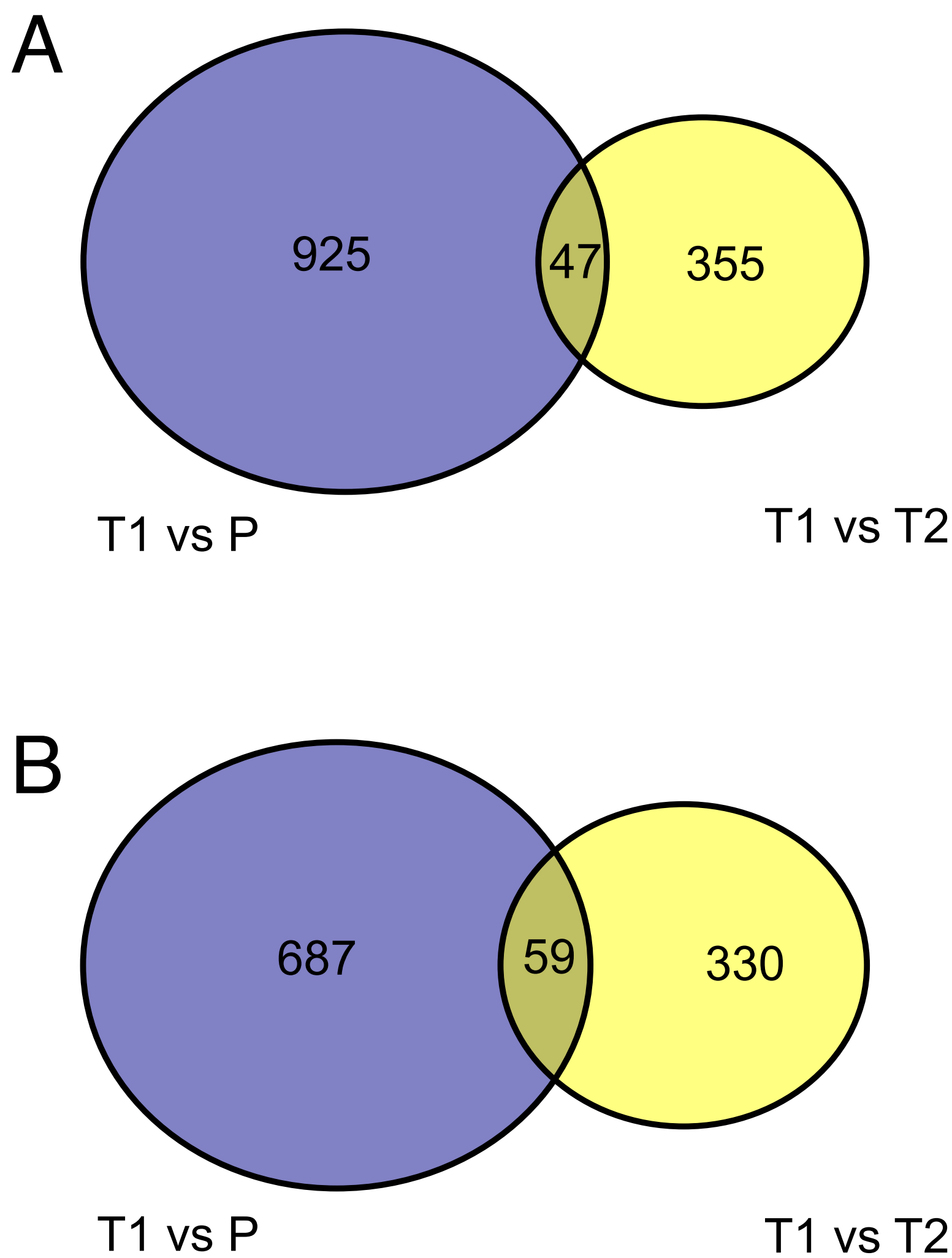

Figure S1: Gene expression unique to T1 legs. (A) Upregulated and **(B)** downregulated genes from differential gene expression analyse comparing T1 legs versus Palps (blue) and T1 legs versus T2 legs (yellow). Genes which are upregulated and downregulated in T1 in both comparisons (47 and 59 respectively) are shown in the overlap.

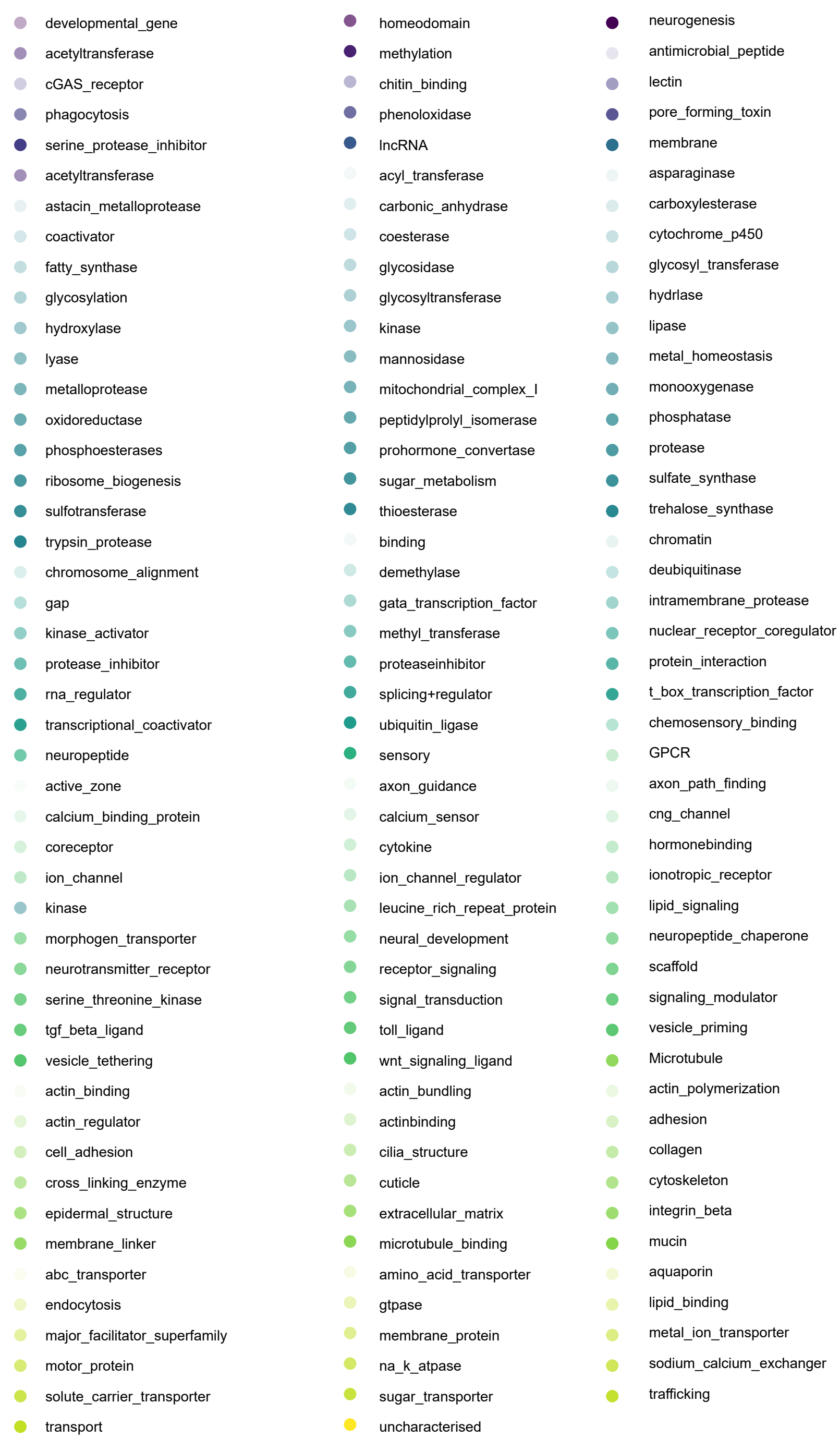

Figure S2: Functional categories Specialised functions of different categories of genes observed as upregulated and differentially expressed in both T1 and Palp when compared with T2, as displayed in Figure 2.

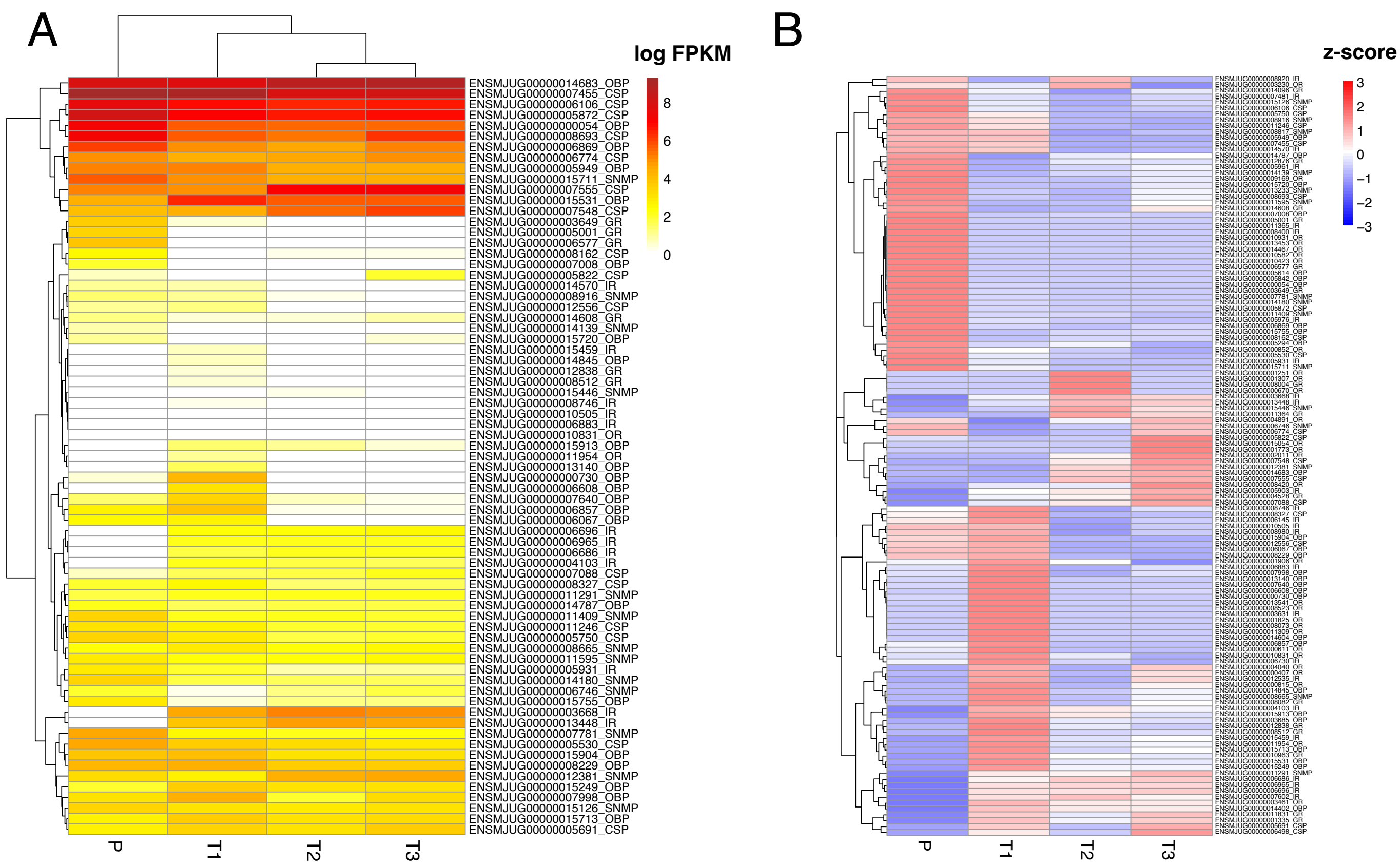

Figure S3: Chemosensory gene expression in all tissues. Expression values given as FPKM in log10 **(A)** and z-scores **(B)** for all annotated chemosensory genes in Palp, T1, T2, and T3 legs. Gene identification numbers and their corresponding gene type are given at the end of each row.

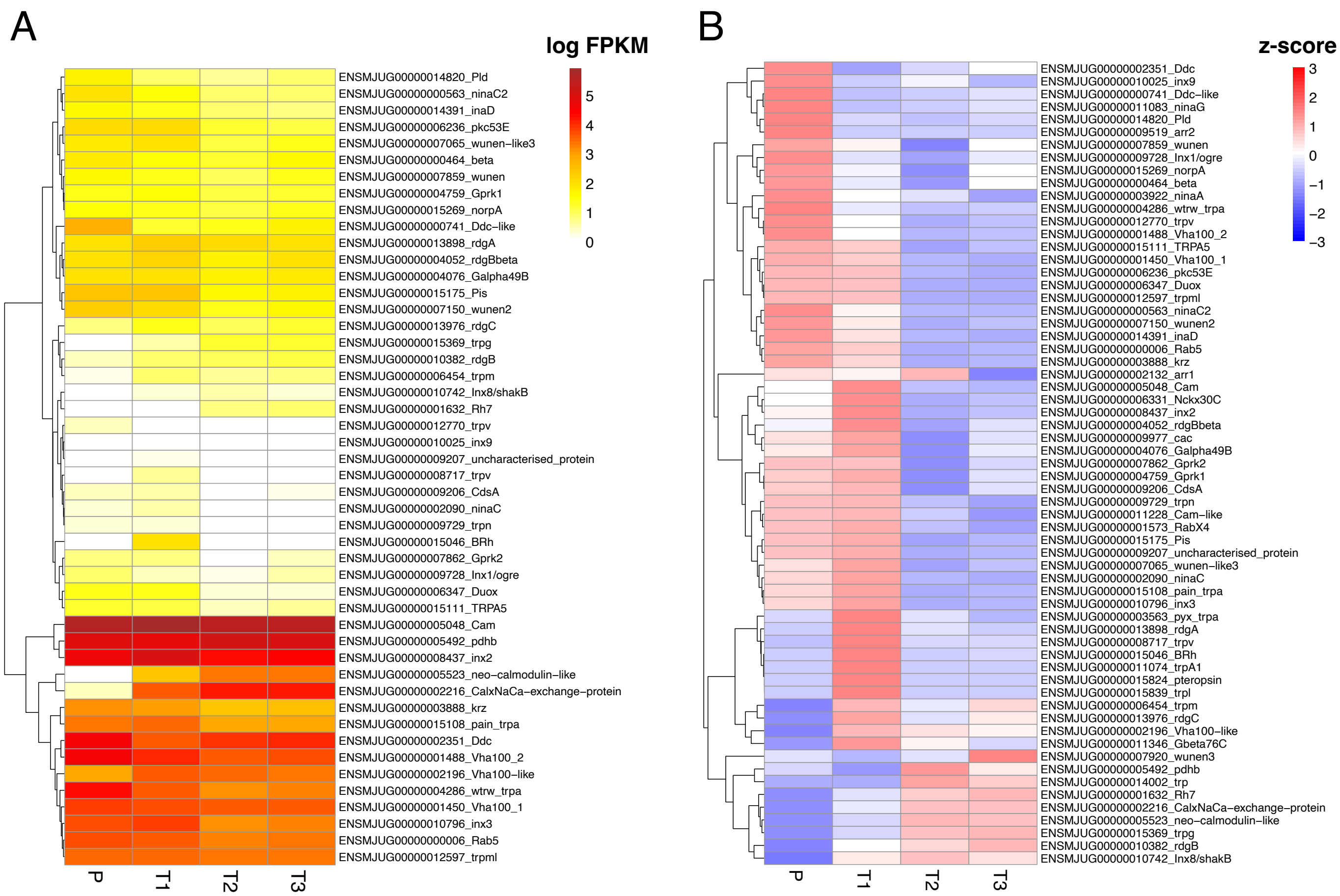

Figure S4: Phototransduction gene expression in all tissues. Expression values given as FPKM in log10 **(A)** and z-scores **(B)** for all annotated phototransduction genes in Palp, T1, T2, and T3 legs. Gene identification numbers and their corresponding gene name are given at the end of each row.

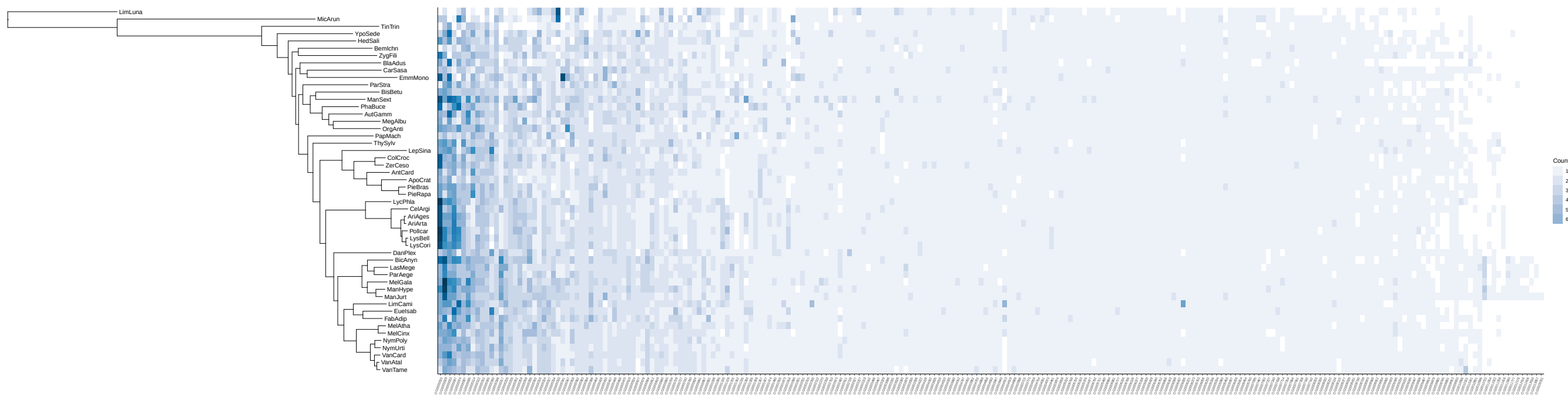

Figure S5: Gene copy number of 277 palp and T1 enriched genes. Species tree of lepidopteran species used in this study. Heatmap to the right corresponds to the copy number of the orthogroups ordered by number of gene copies present from left to right. These orthogroups contain genes in *Maniola jurtina* which are upregulated in palps and T1 legs relative to T2 legs.

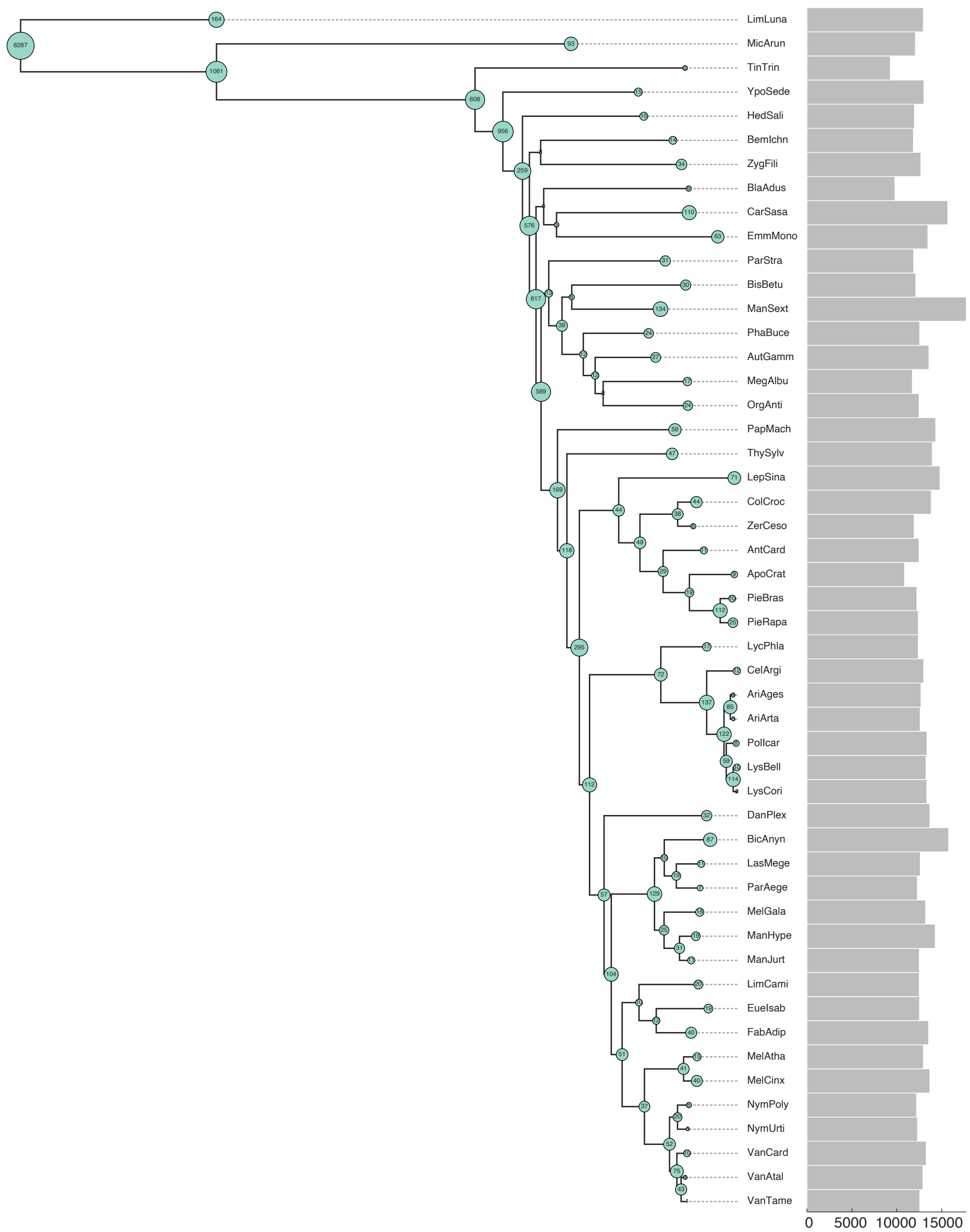

Figure S6: Gene gain across Lepidoptera phylogeny. Species tree of Lepidoptera used in this study with nodes showing the number of orthogroups gained on each branch (as shown by number and scaled green circles). Bar chart of the right shows the number of orthogroups containing each corresponding species.
